# Supplementary material for: A high-strength silicide phase in a stainless steel alloy designed for wear-resistant applications
Source: Nat Commun. 2018 Apr 10;9:1374. doi: 10.1038/s41467-018-03875-9 (PMC5893616; doi:10.1038/s41467-018-03875-9)
Supplement: Supplementary file 1 — Supplementary Information [file 41467_2018_3875_MOESM1_ESM.pdf]

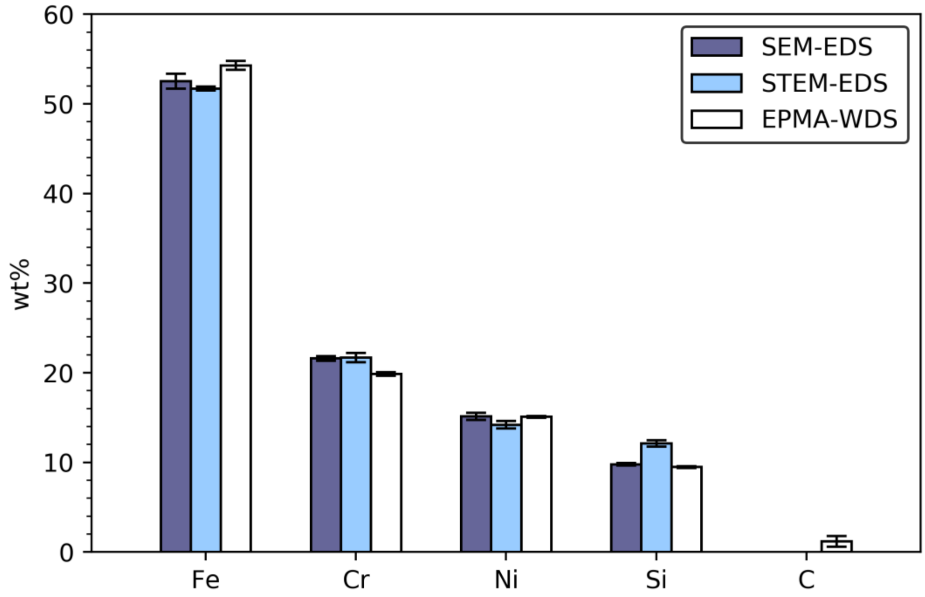

Supplementary Figure 1 | Average point analysis composition results for the π-ferrosilicide phase using SEM-EDS, STEM-EDS and EPMA-WDS techniques. Errors shown are the standard uncertainty of the mean.

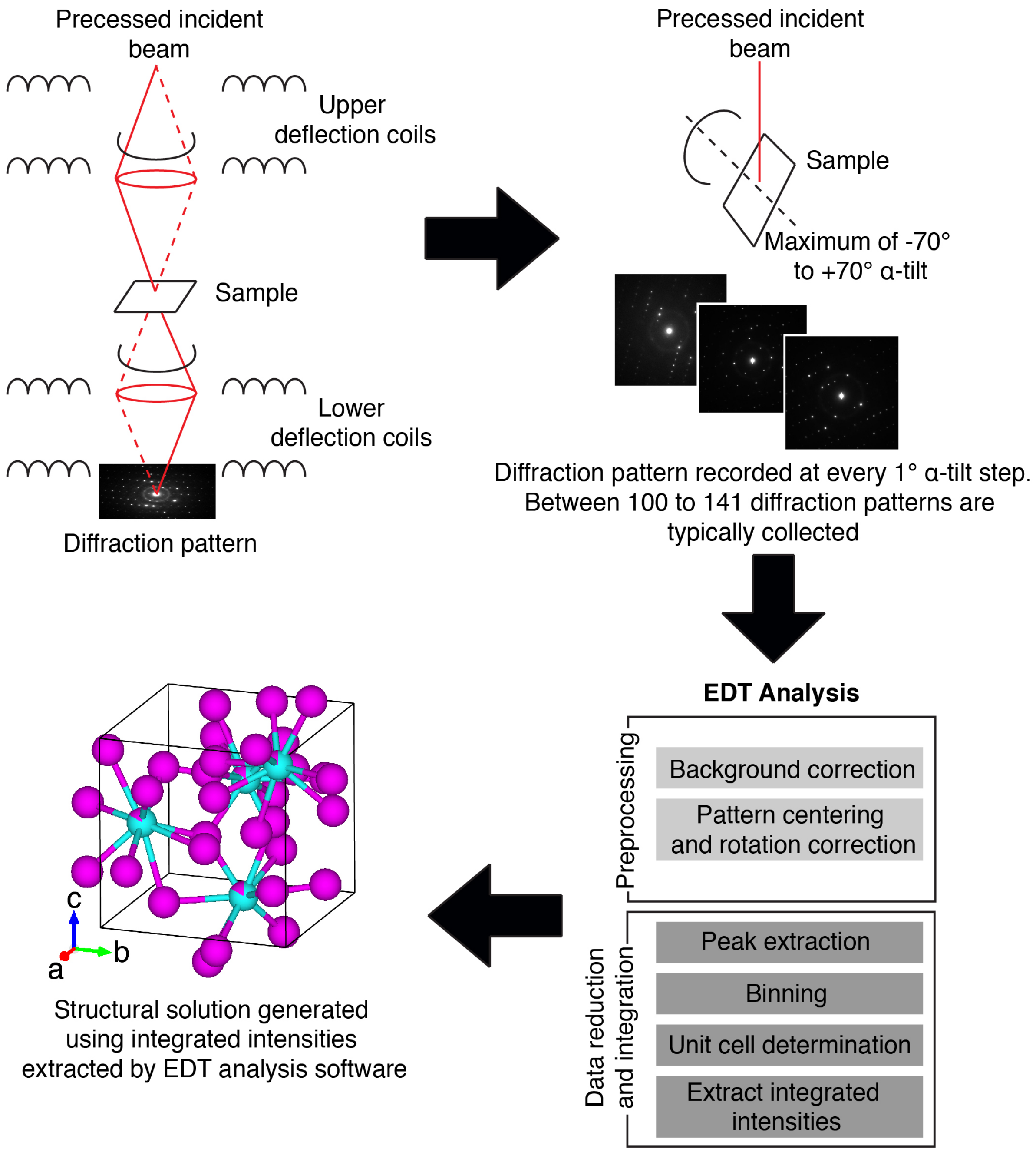

Supplementary Figure 2 | Schematic outlining the process of collecting a tilt-series in the TEM from a single crystal, through to reconstructing the unit cell using EDT analysis software such as using JANA 2006 or ADT3D, allowing a complete structural solution to be generated. A resultant schematic of the unit cell is produced using VESTA V3.4.

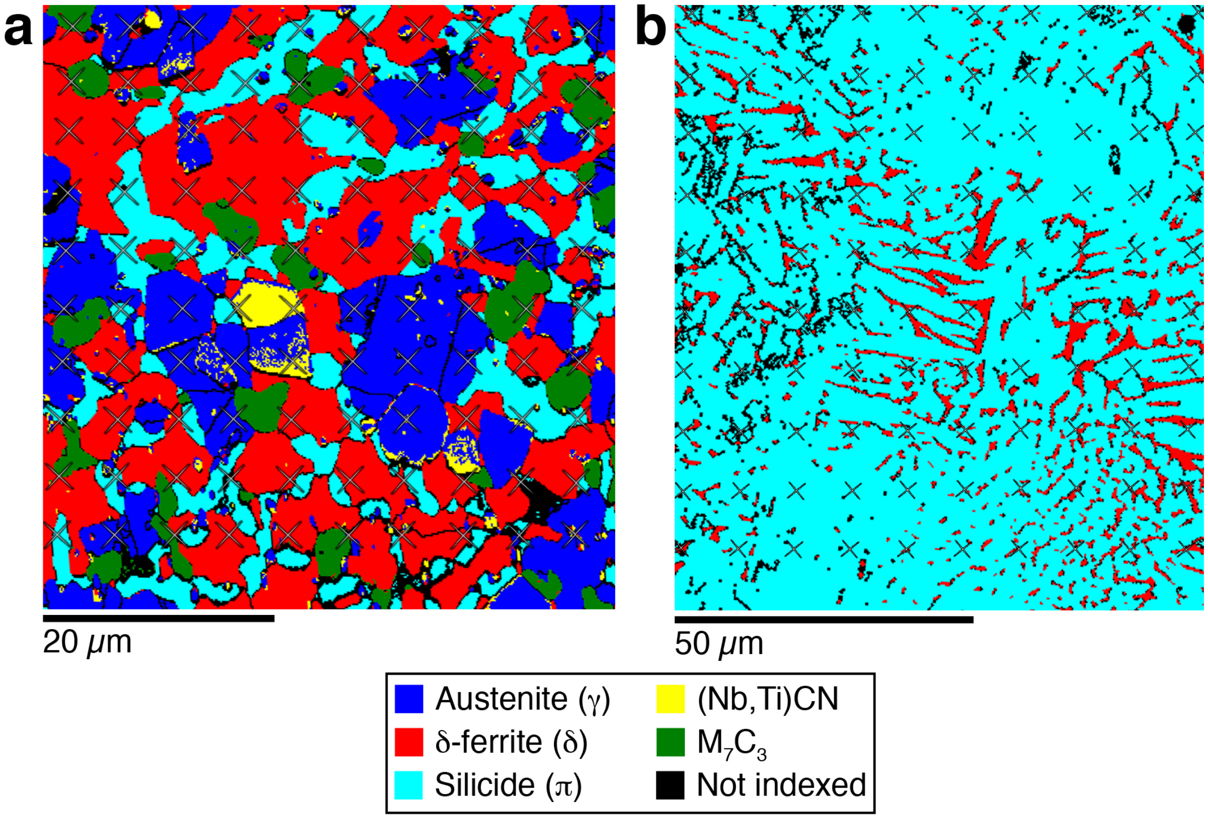

Supplementary Figure 3 | EBSD phase maps showing nanoindent locations across a region of the RR2450 alloy and carbon-deficient silicide phase ingot. a) 45 x 45 μm nanoindent grid locations across a typical region of the RR2450 alloy; b) 90 x 90 μm nanoindent grid across a typical region of the carbon-deficient silicide phase ingot. As the indents are approximately 200 nm across, indent locations on both maps have been marked with crosses to enhance clarity.

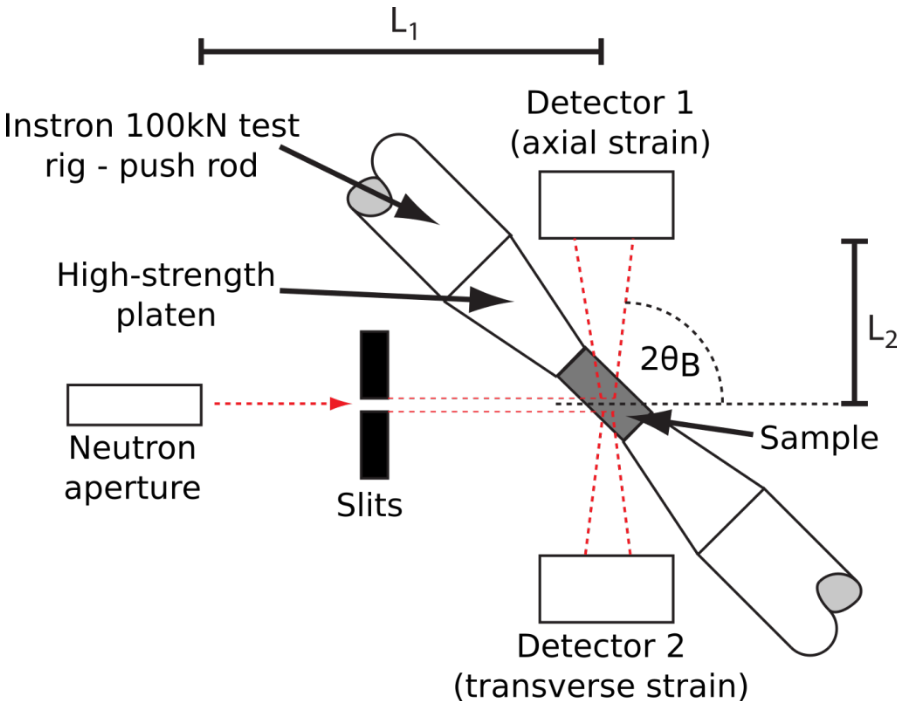

Supplementary Figure 4 | Schematic showing the typical arrangement of the neutron diffraction and in situ compression study carried out on the RR2450 alloy.

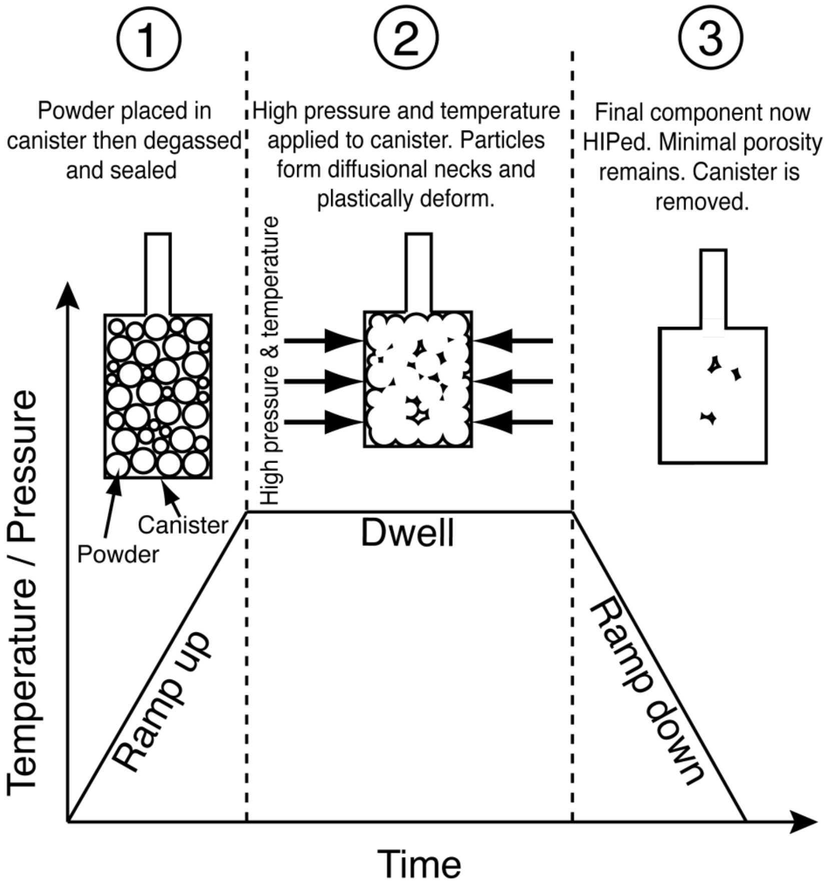

Supplementary Figure 5 | Schematic overview of a typical HIP cycle profile. This can be described in three stages; ramp-up, dwell and ramp-down. This same type of profile was used to produce HIP consolidated RR2450.

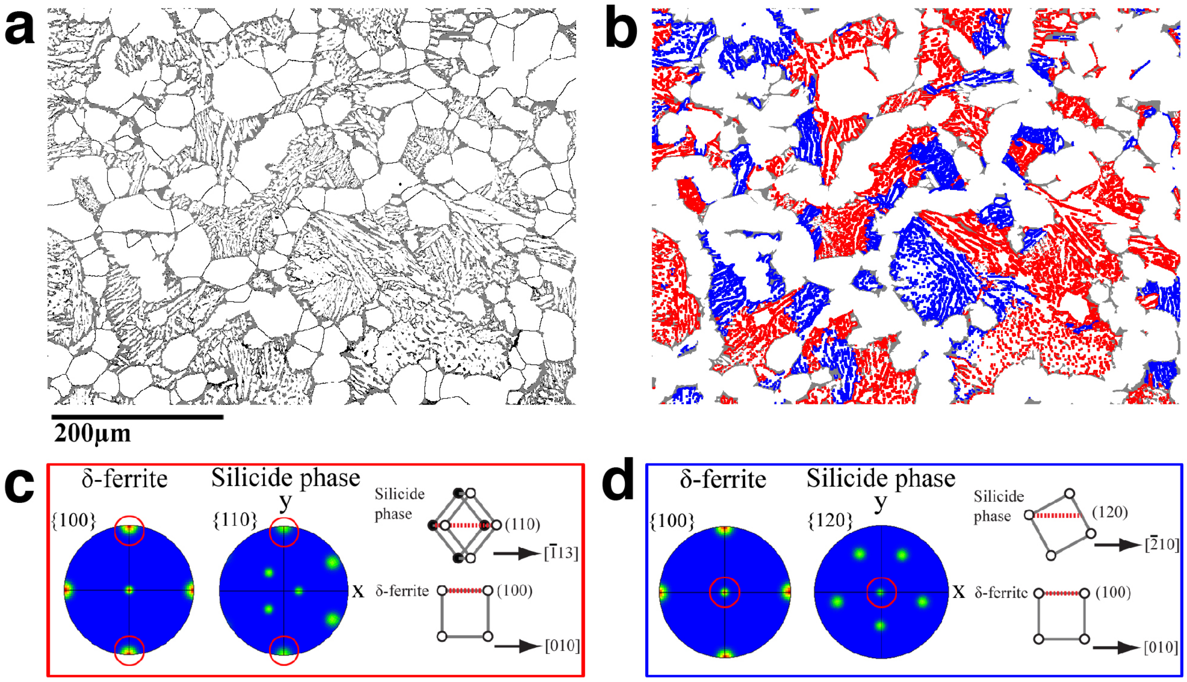

Supplementary Figure 6 | Observed orientation relationship (OR) between ferrite and π-ferrosilicide from EBSD data. a) phase map of the cast carbon-deficient π-ferrosilicide ingot where ferrite grains are shaded grey and π-ferrosilicide grains are shaded white; b) the two identified orientation relationships are shown on the same phase map, grain boundaries are removed for clarity. The colours used correspond to; c) red – the $\{100\}_\delta||\{110\}_\pi$, $\langle 010\rangle_\delta||\langle \bar{1}13\rangle_\pi$ OR showing pole figures from neighbouring ferrite and π-ferrosilicide grains and a schematic representation of the OR; d) blue – the $\{100\}_\delta||\{120\}_\pi$, $\langle 010\rangle_\delta||\langle \bar{2}10\rangle_\pi$ OR showing pole figures from neighbouring ferrite and π-ferrosilicide grains and corresponding OR schematic. Growth directions for each OR are indicated with arrows in the schematic in (c) and (d).

Supplementary Table 1 | Typical compositions (wt% / at%) of the RR2450 and Tristelle 5183 alloys.

Alloy	Fe	Cr	Ni	Nb	Si	C	Ti
RR2450	53.4/48.5	21.0/20.5	9.0/7.8	8.5/4.6	5.8/10.5	1.8/7.6	0.5/0.5
Tristelle 5183	54.6/49.7	21.0/20.5	10.0/8.7	7.5/4.1	5.0/9.0	1.9/8.0	-

Supplementary Table 2 | Phase fractions (%) as determined by laboratory XRD of the HIPed RR2450 alloy, its parent, Tristelle 5183 and the cast carbon-deficient silicide phase ingot.

Material	γ	δ-ferrite	Silicide phase	(Nb,Ti)CN	M_7C_3
Tristelle 5183	50	23	8	7	12
HIPed RR2450 Alloy	27	29	23	8	13
Cast carbon-deficient silicide phase ingot	-	17	83	-	-

Phase fractions marked as '-' were not present.

Supplementary Table 3 | EDT parameters, residuals and crystallographic data for the carbon-containing and carbon-deficient π-ferrosilicide phases with a dynamical refinement carried out.

	π-ferrosilicide (with carbon)	π-ferrosilicide (carbon deficient)
**Crystal data**		
Chemical formula	$(Fe,Ni,Cr)_{4.18}Si_{0.82}$	$(Fe,Ni,Cr)_{3.92}Si_{1.08}$
Crystal system, space group	cubic, $P2_13$	cubic, $P2_13$
a (Å), V(Å^3)	6.1908(1), 237.269(11)	6.1669(2), 234.531(13)
Z	4	4
Calculated density (g.cm^{-3})	7.18	7.06
**Data collection**		
Radiation type	electrons, 120 kV	electrons, 120 kV
Wavelength (Å)	0.0335	0.0335
Precession angle (deg.)	0.5	1.0
Projected crystal dimensions (nm^2)[1]	500x160	126000 (400nm beam)
Average crystal thickness (refined, nm)	30	35
Resolution (Å)	0.714	0.714
Tilt step (deg.)	1	1
No of frames	100	121
Completeness	100%	100%
No. of used reflections (obs/all)	1327/1323	2951/2920
**Dynamical structure refinement**		
g_{max}, S_g^{max}(matrix), R_{Sg}, N_{or}	1.5, 0.01, 0.6, 96	1.5, 0.01, 0.6, 96
R1, wR(F) (obs/all)	6.78/7.03, 9.19/9.20	5.24/5.27, 7.67/7.68
Goodness of fit	2.26	1.67
No. of refined parameters[2]	17+100	17+111

[1] In case of the carbon-deficient silicide the size of the illuminated part of the crystal was defined by the size of the illuminating beam.

[2] Number of refined parameters is split into scale factors for individual frames and the number of actual structural parameters like atomic coordinates, displacement parameters and occupancy factors.

Supplementary Table 4 | Atomic coordinates, thermal parameters and occupancy of the RR2450 (carbon containing) π-ferrosilicide phase, determined by dynamical refinement.

**Atom**	**x**	**y**	**z**	**Ueq (Å²)¹**	**Occ.**	**Wyckoff**
Fe1	0.18647(13)	0.68647(13)	0.81353(13)	0.0055(2)	1	4a
Fe2	0.37684(16)	0.70335(15)	0.45210(15)	0.0150(3)	1	12b
Si3	0.9374(2)	0.9374(2)	0.9374(2)	0.0048(5)	0.82(2)	4a
Fe3	0.9374(2)	0.9374(2)	0.9374(2)	0.0048(5)	0.18(2)	4a

[1]Ueq is obtained from anisotropic displacement parameters as: $U_{eq} = \frac{1}{3}\sum\sum U_{ij} a_i^* a_j^* \boldsymbol{a}_i . \boldsymbol{a}_j$.

Supplementary Table 5 | Independent interatomic distances of the RR2450 (carbon-containing) π-ferrosilicide phase calculated using dynamical refinement.

**Atom 1**	**Atom 2**	**Distance (Å)**
Fe1	Fe2	2.5311(12)
Fe1	Fe2	2.5910(12)
Fe1	Fe2	2.6161(13)
Fe1	Si3	2.3192(15)
Fe2	Fe2	2.5927(14)
Fe2	Fe2	2.6010(13)
Fe2	Fe2	2.6125(13)
Fe2	Si3	2.5055(16)
Fe2	Si3	2.6388(16)
Fe2	Si3	2.5911(16)

Supplementary Table 6 | Atomic coordinates, thermal parameters and occupancy of the cast (carbon deficient) π-ferrosilicide phase, determined by dynamical refinement.

**Atom**	**x**	**y**	**z**	**Ueq (Å²)[1]**	**Occ.**	**Wyckoff**
Fe1	0.93671(9)	0.93671(9)	0.93671(9)	0.00450(17)	0.875(13)	4a
Si2	0.93671(9)	0.93671(9)	0.93671(9)	0.00450(17)	0.125(13)	4a
Fe2	1.12664(11)	1.29708(10)	0.95294(10)	0.0174(2)	1	12b
Si1	1.18724(15)	0.68724(15)	0.81276(15)	0.0068(3)	0.954(14)	4a
Fe3	1.18724(15)	0.68724(15)	0.81276(15)	0.0068(3)	0.046(14)	4a

[1]Ueq is obtained from anisotropic displacement parameters as: $U_{eq} = \frac{1}{3}\sum\sum U_{ij} a_i^* a_j^* \boldsymbol{a}_i . \boldsymbol{a}_j$.

Supplementary Table 7 | Independent interatomic distances of the cast (carbon-deficient) π-ferrosilicide phase calculated using dynamical refinement.

**Atom 1**	**Atom 2**	**Distance (Å)**
Fe1	Fe2	2.5141(8)
Fe1	Fe2	2.5823(8)
Fe1	Fe2	2.6105(8)
Fe1	Si1	2.3104(11)
Fe2	Fe2	2.5993(9)
Fe2	Fe2	2.5783(9)
Fe2	Fe2	2.5977(9)
Fe2	Si1	2.5001(11)
